# Effect of Androgen Blockade on HER-2 and Matrix Metalloproteinase-2 Expression on Bone Marrow Micrometastasis and Stromal Cells in Men with Prostate Cancer

**DOI:** 10.1155/2013/281291

**Published:** 2013-05-14

**Authors:** N. P. Murray, E. Reyes, L. Badinez, N. Orellana, C. Fuentealba, R. Olivares, J. Porcell, R. Dueñas

**Affiliations:** ^1^Hospital de Carabineros of Chile, Simón Bolívar 2200 Ñuñoa, 7770199 Santiago, Chile; ^2^Circulating Tumor Cell Unit, Faculty of Medicine, University Mayor, Renato Sánchez 4369, Las Condes, 27550224 Santiago, Chile; ^3^Institute of Bio-Oncology, Avenida Salvador 95, Oficina 513, Providencia, 7500710 Santiago, Chile; ^4^Universidad Diego Portales, Manuel Rodriguez Sur 415, 8370179 Santiago, Chile; ^5^Foundation of Arturo López Pérez, Rancagua 878, Providencia, 7500921 Santiago, Chile

## Abstract

*Introduction*. HER-2 has been associated with castrate resistant prostate cancer and matrix metalloproteinase-2 (MMP-2) in the dissemination and invasion of tumor cells as well as activating angiogenesis. We present an immunocytochemical study of the effect of androgen blockade on the expression of HER-2 and MMP-2 in bone marrow micrometastasis and the surrounding stromal cells in men with prostate cancer. *Methods and Patients*. A cross-sectional study of men with prostate cancer. Touch preps were obtained from bone marrow biopsies of men with prostate cancer, before and after radical prostatectomy and during androgen blockade. Micrometastasis detected with anti-PSA immunocytochemistry underwent processing with anti-HER-2 and anti-MMP-2 immunocytochemistry. Patients were defined as HER-2 positive or negative, MMP-2 negative or an MMP-2 pattern described as border or central and stromal MMP-2 defined as positive or negative. The expression of the biomarkers was compared before and after primary treatment and during androgen blockade in relation to the serum PSA at the time of sampling and duration of androgen blockade. *Results*. 191 men participated, 35 men before surgery and 43 after surgery; there were no significant differences in HER-2 expression between groups, there was no MMP-2 expression centrally or stromal expression of MMP-2. In men with androgen blockade, HER-2 expression was significantly higher; there was a trend for increasing HER-2 expression up to 5 years; central MMP-2 expression significantly increased after 3 years, while stromal MMP-2 significantly increased after 6 years. MMP-2 expression both in micrometastasis and stroma was significantly associated with HER-2 expression. Expression of MMP-2 at the border of the micrometastasis was not associated with HER-2 expression and occurred in the absence of androgen blockade. *Conclusions*. Androgen blockade decreases serum PSA by eliminating HER-2 negative prostate cancer cells. However, there is early selection of HER-2 positive cancer cells which leads to androgen independence and to increased expression of MMP-2 activity in the micrometastasis. The increased MMP-2 activity in the micrometastasis increases the expression of MMP-2 in the surrounding stromal cells and thus could promote angiogenesis and tumor growth resulting in macrometastatic androgen independent disease.

## 1. Introduction

Since the publication of Huggin's work in 1942 [1], androgen blockade has been a treatment option for metastastic prostate cancer and for biochemical failure after primary therapy. Early-stage prostate cancer exhibits androgen dependence, and as a result of androgen blockade or withdrawal, prostate cancer cells undergo cell cycle arrest or apoptosis. Although most patients respond initially to therapy, these patients eventually relapse and die from their disease [2]. Once hormone refractory disease is present, the prognosis is very poor with a median survival of 9–12 months [3]. The mechanisms responsible for the initial survival and subsequent proliferation of castrate resistant prostate cancer cells remain poorly characterized. Therefore, identification of patients who are likely to fail androgen blockage would be helpful for selecting patients who are best suited for other treatments or clinical trials of early systemic intervention [4].

One such biomarker is HER-2, which is a member of the ErbB family of receptor tyrosine kinases and plays a crucial role in growth, differentiation, and motility of normal and cancer cells. HER-2 has been proposed as a survival factor for prostate cells in the absence of androgens, possibly by activating the androgen receptor [5–7]. In hormone-naive patients, whether in patients undergoing observation or posttreatment with or without biochemical failure, the expression of HER-2 was infrequent both in CPCs and micrometastasis, whereas patients treated with androgen blockage had a significantly increased levels of HER-2 expression in both CPCs and micrometastasis [8]. Inhibition of HER-2 protein suppresses HER-2/PI3K/Akt pathway signaling with subsequent suppression of proteolytic activity by downregulating the activity of metalloproteinases [9]. Matrix metalloproteinase-2 (MMP-2) expression in primary prostate cancer is associated with a worse prognosis [10–12]. MMP-2 is thought to be important in the dissemination and invasion of cancer cells [13, 14] and through the activation of MMP-9 thought to activate angiogenesis and thus permits tumor growthand the formation of metastasis [15].

We present a cross-sectional cohort study of the expression of HER-2 and MMP-2 in bone marrow micrometastasis detected in bone marrow biopsies and compare the effect of androgen blockade on HER-2 and MMP-2 expressions in tumor and surrounding stromal cells with that of men without androgen blockade. We hypothesize that micrometastasis from higher grade tumors or those micrometastases exposed previously to androgen blockade have a higher expression of HER-2 protein, and this in turn leads to higher MMP-2 expression in tumor cells and stromal cells which finally leads to angiogenesis and macrometastasis formation.

## 2. Patients and Methods

Men diagnosed with prostate cancer attending the Hospital de Carabineros de Chile and Instituto de Bio-Oncología, Santiago, Chile, between 2008 and 2011 were asked to participate in the study. Patient records were used to retrieve clinical information (age, stage, Gleason score, length of treatment with androgen blockade where appropriate, bone scan results, serum PSA, and time from diagnosis at the time of sampling).

**Figure 1:**
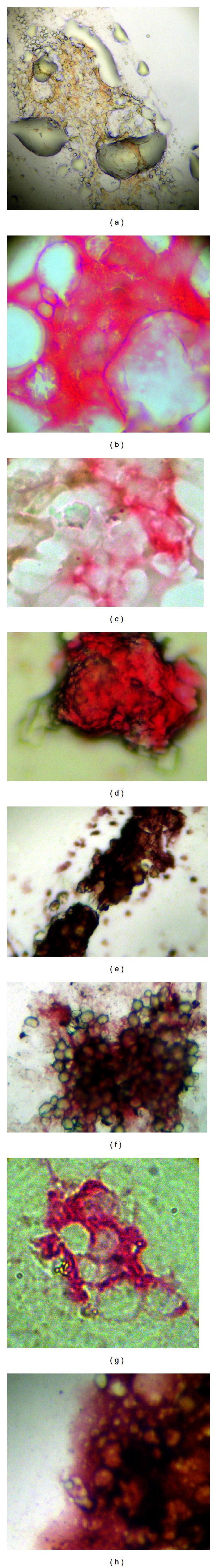
(a) mM negative, (b) mM positive, (c) MMP-2 negative, (d) MMP-2 border positive, (e) MMP-2 positive, stroma negative, (f) MMP-2 positive, stroma positive, (g) HER-2 negative, and (h) HER-2 positive.

The criteria of ISHAGE were used to evaluate immunostained cells [16], and mM defined as PSA staining cells was detected in bone marrow fragments from biopsy specimens (Figures 1(a) and 1(b)).

### 2.1. Inclusion Criteria

These include (a) biopsy proven prostate cancer; (b) written informed consent; (c) with or without androgen blockade; and (d) negative bone scan within three months of the sampling.

### 2.2. Sample Preparation

A bone marrow biopsy was taken from the posterior superior iliac crest. The bone marrow biopsy sample was used to make 4 “touch preps” using silanized slides (DAKO, USA). The slides were air-dried for 24 hours, finally fixed in a solution of 70% ethanol, 5% formaldehyde and 25% PBS for 5 minutes, and then washed 3 times with PBS.

### 2.3. Immunocytochemistry

Monoclonal antibodies directed against PSA clone 28A4 (Novacastra, UK) in a concentration of 2,5 *μ*g/mL were used to detect prostate cells and identified using a detection system based on alkaline phosphatase-antialkaline phosphatase (LSAB2 DAKO, USA) with new fuchsin as the chromogen, according to the manufacturers' instructions. To permit the rapid identification of positive cells there was no counterstaining with Mayer's hematoxylin. Leisamvole (DAKO, USA) was used as an inhibitor of endogenous alkaline phosphatase. Positive (prostate) and negative (colon) controls were processed in the same way.

Positive samples underwent a second stage, half being used to detect MMP-2 expression and the other half HER-2 expression.

#### 2.3.1. MMP-2 Expression

Samples were incubated for 1 hour at room temperature with anti-MMP-2 clone 1B4 (Novocastra, UK) and identified with a system of detection based on peroxidase (LSAB2, DAKO, USA) with DAB (DAKO, USA) as the chromogen, according to the manufacturers' instructions. Endogenous peroxidase was inhibited using an inhibitor (DAKO, USA) according to the manufacturer's instructions.

*Definition of Expression of MMP-2*. The criterion to define a cell expressing MMP-2 was that of Trudel et al. (2003) [10], micrometastasis being defined as positive or negative, with a central or border pattern of expression (see Figures 1(c), 1(d), and 1(e)). In the samples of bone marrow biopsy touch preps the expression of MMP-2 in the surrounding non-PSA expressing cells was analyzed. The expression of MMP-2 in these cells was noted as present or absent (see Figures 1(e) and 1(f)).

#### 2.3.2. HER-2 Expression

HER-2 expression was determined using the HercepTest, according to manufacturer's instructions. Cells were classified as PSA positive and HER-2 either negative or positive and with the score 0–3+ regarding HER-2 staining intensity (Figures 1(g) and 1(h)). HER-2-positive patients were defined according to the criteria of Osman et al. [6] as 2+ and 3+ staining in more than 10% of PSA-positive cells. A mean expression of HER-2 per cell was calculated using the following formula: sum of HER-2 scores/number of cells counted.

Samples were analyzed at low power, and photographed at a magnification of 400x using a digital camera, Samsung Digimax D73, and processed with the Digimax program for Windows 98. The immunocytochemical evaluation was performed by a single person, blinded to the clinical details using a coded system.

The patients were divided into 3 groups:

- preradical prostatectomy and bone scan negative: patients with micrometastasis were analyzed for MMP-2 and HER-2 expressions,
- postradical prostatectomy bone scan negative without evidence of biochemical failure, defined as a PSA > 0.2 ng/mL and without androgen blockade: patients were analyzed for MMP-2 and HER-2 expressions, and
- postradical prostatectomy, biochemical failure, and with androgen blockade. Patients were analyzed for MMP-2 and HER-2 expressions, according to serum PSA at the time of sampling and time elapsed from starting of androgen blockade.

### 2.4. Statistical Analysis

Descriptive statistics were used for demographic variables, expressed as mean and standard deviation in the case of continuous variables with a normal distribution. In case of an asymmetrical distribution the median and interquartile range (IQR) values were used. Noncontiguous variables were presented as frequencies. The Student's *t*-test was used to compare continuous variables with a normal distribution, Chi-squared, Kruskal-Wallis, and log regression for the differences in frequency. The kappa test was used for tests of concordance.

The analysis was firstly to compare the expressions of HER-2 and MMP-2 in the micrometastasis and stromal expression of MMP-2 in men with and without androgen blockade and secondly, in the men undergoing androgen blockade, to compare the expressions of HER-2 and MMP-2 in the micrometastasis and stromal expression of MMP-2 with the serum PSA at the time of sampling and with the length of androgen blockade at the time of sampling.

### 2.5. Ethical Considerations

The study was directed with complete conformity with the principles of the declaration of Helsinki and approval of the local ethical committees.

## 3. Results

A total of 191 men participated in the study.

### 3.1. Preprostatectomy Radical

35 men with a mean age of 70.0 ± 10.5 years and a median serum PSA of 4.83 ng/mL (IQR 3.03–11.48 ng/mL) comprised the group. Overall micrometastasis was detected in 26/35 (74.3%); there were significantly fewer micrometastases detected in patients with Gleason 4 and stage 1 cancer (Table 1).

MMP-2 expression was seen in 3/26 (11.5%) of micrometastases, at the edges of the bone marrow fragments; corresponding to Gleason 9 and two Gleason 7 patients, there was no centrally distributed MMP-2 expression or stromal expression of MMP-2. HER-2 expression was defined as positive in 4/26 (15.4%) of patients, with an average expression of 0.21 ± 0.16 per cell. HER-2 expression was not associated with Gleason score or stage, corresponding to 2 patients with Gleason 5, 1 with Gleason 6, and 1 with Gleason 9 and 2 patients with stage 2 and 2 patients with stage 3 disease. 12/26 had no HER-2 expression detected.

### 3.2. Postprostatectomy Radical without Evidence of Biochemical Failure or Previous Androgen Blockade

43 men with an average age of 71.1 ± 9.0 years and a median serum PSA of 0.04 ng/mL (IQR 0.02–0.10 ng/mL) comprised the group. Micrometastases were detected in 28/43 (65.1%) of patients. There were no differences in the frequency of micrometastases detection according to Gleason score, but stage 2 patients had significantly fewer micrometastases detected than stage 3 patients (Chi-squared *P* = 0.04) (see Table 2). There was no significant difference in the frequency of micrometastasis detection between pre- and postradical prostatectomy groups (Chi-squared *P* = 0.38).

MMP-2 expression was seen in 2/43 (4.7%) of micrometastases, at the edges of the bone marrow fragments; corresponding to 2 Gleason 9 patients, there was no centrally distributed MMP-2 expression or stromal expression of MMP-2. HER-2 expression was defined as positive in 7/43 (16.3%) of patients, with an average expression of 0.30 ± 0.21 per cell. HER-2 expression was not associated with Gleason score or stage, corresponding to 2 patients with Gleason 5, 2 with Gleason 6, 1 with Gleason 7, and 1 with Gleason 9 and in 2 patients with stage 2 and 5 patients with stage 3 disease. 22/43 had no HER-2 expression detected.

*Comparison between Men Pre- and Postradical Prostatectomy and without Androgen Blockade*. There were no significant differences between the groups in terms of frequency of micrometastases detected, frequency and pattern of MMP-2 expression, or frequency of HER-2 expression.

### 3.3. Postprostatectomy Radical with Biochemical Failure and Androgen Blockade

113 men with a mean age of 73.1 ± 8.4 years and a median serum PSA of 1.70 ng/mL (IQR 0.70–9.09 ng/mL) formed the group. Micrometastases were detected in 87/113 (77.0%) of cases, and such cases represented systemic failure of primary treatment and systemic therapy with androgen blockade. Central expression of MMP-2 was detected in 21/87 (24.1%) of micrometastases, and HER-2 expression was positive in 41/87 (47.1%) of micrometastases. Stroma expressing MMP-2 was detected in 14/87 (16.1%) of cases. Comparing men with androgen blockade with those without androgen blockade (combined pre- and postprostatectomy) the frequency of HER-2-positive patients was significantly higher (*P* = 0.0004 Chi-squared) in men treated with androgen blockade as was the frequency of central MMP-2 expression (*P* = 0.005 Chi-squared). Mean HER-2 expression per cell was significantly higher compared with men without androgen blockade 1.28 ± 0.53 versus 0.21 ± 0.16 and 0.30 ± 0.21, respectively (*P* < 0.03).

#### 3.3.1. Comparison between Central Expression of MMP-2 and HER-2 Expression in Micrometastasis

There was a significant association between the coexpression of MMP-2 and HER-2 in bone marrow micrometastasis (*P* = 0.005 Chi-squared) (Table 3).

#### 3.3.2. Comparison between Serum PSA and MMP-2 and HER-2 Expressions 

We analyzed the relation between the serum PSA at the time of sampling and the frequency of MMP-2 and HER-2 expressions. The inference was that the higher the serum PSA, the more advanced the disease. Based on a pilot study we arbitrarily divided the group into 3 subgroups, those with a serum PSA < 2.0 ng/mL, those with a serum PSA of 2.0–10.0 ng/mL, and those with a PSA > 10.0 ng/mL (Table 4).

The frequency of MMP-2 expression was significantly higher in men with a serum PSA > 10.0 ng/mL than in men with a serum PSA of < 2.0 ng/mL or 2.0–10.0 ng/mL (*P* = 0.0006 and *P* = 0.008 Chi-squared, resp.). Chi-squared for trend analysis was positive, *P* > 0.00001, with an overall risk of 1.00, 4.00, and 23.2, respectively.

The frequency of HER-2 expression was not significantly different between the three groups, <2.0 ng/mL versus 2–10 ng/mL (*P* = 0.20 Chi-squared), <2.0 versus >10.0 ng/mL (*P* = 0.07 Chi-squared), and 2–10 ng/mL versus >10.0 ng/mL (*P* = 0.94 Chi-squared). In the analysis for trends *P* = 0.036 Chi-squared with an overall risk of 1.00, 1.73, and 2,14; there was a tendency for higher HER-2 expression with increasing serum PSA levels.

The combined expression MMP-2 (+) HER-2 (+) increased with increasing serum PSA (*P* = 0.0007 Chi-squared for trends, OR 1.00, 1.81, and 9.50, resp.); similarly the expression MMP-2 (−) HER-2 (−) decreased with increasing serum PSA (*P* = 0.012 Chi-squared for trends, OR, 1.00, 0.33, and 0.29, resp.).

#### 3.3.3. Comparison between Exposure Time to Androgen Blockade and Expression of MMP-2 and HER-2

We divided the patients into 4 subgroups based on the time exposed to androgen blockade, 0–2 years, 3–5 years, 6–10 years, and >10 years, and determined the frequency of the expression of MMP-2 and HER-2 (Table 5).

Analysis for trends (Chi-squared) showed a significant difference in the expression of MMP-2 with time, *P* = 0.0008, with an overall risk of 1.00, 2.29, 4.14, and 5.44 for the different time periods. This suggests that central expression of MMP-2 increases with the period of androgen blockade and is a later event in the process of phenotypic change following androgen blockade.

Analysis for trends did not show a significant difference in the expression of HER-2 with time (*P* = 0.15, Chi-squared), with an overall risk of 1.00, 3.00, 2.00, and 1.50, respectively. This suggests that the increase in HER-2 expression is an early event after the initiation of androgen blockade and remains constant with time.

**Figure 2:**
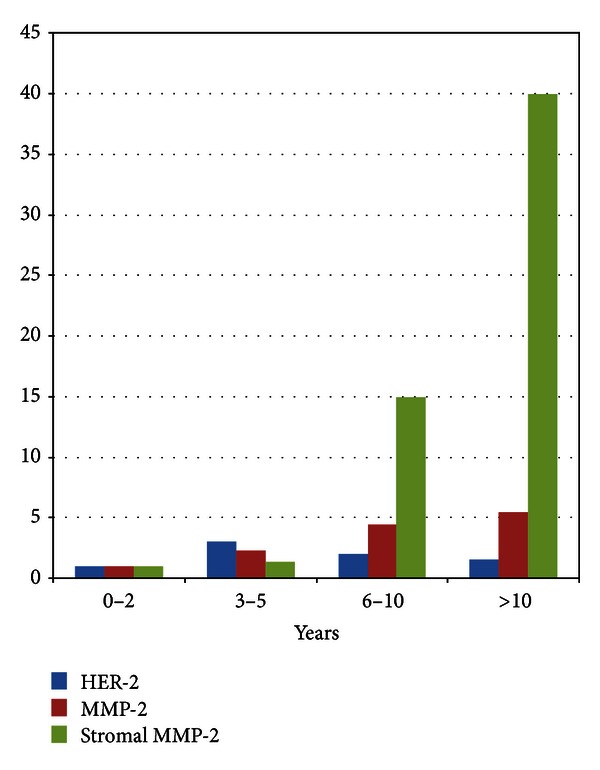
Changes in overall risk for HER-2, MMP-2, and stromal MMP-2 expression with time.

Comparing the frequency of coexpression of MMP-2 (+) and HER-2 (+) there was increased frequency of expression with time (Chi-squared for trends *P* = 0.002, OR 1.00, 10.1, 14.9, and 24.0, resp.); however there was no such trend for MMP-2 (−) HER-2 (−)  (*P* = 0.06 Chi-squared for trends; Figure 2).

#### 3.3.4. Comparison of Stromal MMP-2 Expression

Stromal expression of MMP-2 was compared with that of micrometastasis MMP-2 (Table 6); there was a significant association in the coexpression or absent expression of MMP-2 in stromal and micrometastatic cells.

It can be seen that stromal MMP-2 occurs when there is MMP-2 expression in the micrometastasis; however, the presence of MMP-2 expression in the micrometastasis is not necessarily associated with stromal MMP-2 expression.

Stromal expression of MMP-2 was compared with that of micrometastasis HER-2 expression (Table 7). It can be seen that stromal MMP-2 expression occurs when there is HER-2 expression in the micrometastasis; however the presence of HER-2 expression is not necessarily associated with stromal MMP-2 expression.

#### 3.3.5. Comparison between Serum PSA and Stromal Expression of MMP-2

There was a significant association with the expression of stromal MMP-2 with increasing serum PSA (Table 8; Chi-squared for trends, *P* > 0.00001) with an overall risk of 1.00, 6.50, and 65.6, respectively. 

#### 3.3.6. Comparison between Time Exposed to Androgen Blockade and Expression of Stromal MMP-2

There was significant association of increasing frequency of stromal MMP-2 expression with length of androgen blockade (Table 9; Chi-squared for trend, *P* < 0.00001) with an overall risk of 1.00, 1.33, 14.93, and 192.0, respectively. This suggests that the expression of stromal MMP-2 is a later event and requires years of exposure to androgen blockade.

The changes of the expression of HER-2, MMP-2, and stromal MMP-2 can be represented graphically to more easily show the changes with time. The increased frequency of HER-2 expression is an early event after initiation of androgen blockade; then the expression of MMP-2 in the micrometastasis slowly increases with time, and finally stromal MMP-2 expression increases, the changes in MMP-2 expression being later events.

## 4. Discussion

Over a century ago, Stephen Paget proposed the seed and soil hypothesis, whereby metastasis depends on the cross-talk between selected cancer cells (the “seeds”) and specific organ microenvironments (the “soil”). Fidler has extensively reviewed this hypothesis and concluded that the potential of a tumor cell to metastasize depends on its interactions with the homeostatic factors that promote tumor cell growth, survival, angiogenesis, invasion, and metastasis [17].

Early-stage prostate cancer exhibits androgen dependence, and as a result of androgen blockade or withdrawal, prostate cancer cells undergo cell cycle arrest or apoptosis. The use of androgen blockage, medically or surgically, is the main form of therapy for men with metastatic disease or as adjuvant therapy in high-risk patients. Although most patients respond initially to therapy, these patients eventually relapse and die from their disease [7]. Once castrate resistant disease is present, the prognosis is very poor with a median survival of 9–12 months [8].

HER-2 expression was positive in 16% of hormone naïve patients, similar to data previously published [8] and those found in primary prostate cancers [11]. However to be classified as HER-2 positive, >10% of the cancer cells must express HER-2 with a 2+ or 3+ staining; our results show that many micrometastases are classified as HER-2 negative but contain some HER-2 positive cells. Within 2 years of starting androgen blockade, the frequency of HER-2-positive micrometastasis increased significantly, to a maximum of 60% between 3 and 5 years. This has clinical implications. Pantel et al. [18] have shown that, after neoadjuvant androgen blockade, 16/21 (76%) patients become negative for bone marrow micrometastasis, while a further 4/21 (19%) had decreased numbers of cells detected. Köllermann et al. [19] later demonstrated that patients positive for bone marrow micrometastasis after neoadjuvant androgen blockade had a worse prognosis. It has been reported that, in men with micrometastasis reevaluated after 1 year androgen blockade decreases serum PSA but in HER-2-positive disease does not completely eliminate the micrometastasis [12].

Thus changes in the environment (the “soil”) are capable of selecting HER-2-positive cells, which by their survival implies they are androgen resistant. If HER-2-positive cells survive by activating the androgen receptor, as postulated by some workers [11], this may also stimulate the expression of androgen receptor controlled proteins. In clinical samples, HER-2 expression is elevated in androgen independent tumors [20, 21] and is an early event in the androgen dependence-to-independence switch [22]. The proposed mechanism for the role of HER-2 in hormone escape is that it activates androgen receptor phosphorylation (via the MAPK or AKT pathways) which in turn maintains the androgen receptor integrity and thus its function in the absence of testosterone [23, 24]. In prostate cell models it has been reported that, in androgen independent cell lines, HER-2 expression and AKT activation are increased, and the use of the anti-HER-2 drug trastuzumab can reverse this [25].

In mice models, the deficiency of MMP-2 results in a reduction of immature blood vessels and without neovascularization results in a reduced tumor burden [26]. Our results show that MMP-2 expressed centrally in the micrometastasis increased steadily, with a significant difference from baseline results at 6 years; the tendency was a steady increase with time. We propose that the change in the microenvironment brought about by androgen blockade selects HER-2-positive cells, and thus “the soil” selects “the seed.” This in turn changes “the seed” with increased MMP-2 expression, and would seem from the results to be a later event, following the peak of HER-2 expression. Increased MMP-2 expression would permit the stimulation of angiogenesis, activate the micrometastasis, and permit increased proliferation and growth as suggested by animal models [26].

In cell culture studies androgen stimulates MMP-2 expression, and androgen stimulated pro-MMP-2 expression occurs at the gene transcription level via androgen receptor transactivation and dependent on P13K activity [27]. Both androgen stimulated pro-MMP-2 expression and MMP-2 promoter activity can be abolished by the androgen antagonist bicalutamide [26]. Furthermore, in gastric cancer models HER-2 has been shown to increase the transcription of MMP-1 through the activation of the MMP-1 promoter, and HER-2 knockdown resulted in its downregulation [27]. MMP-1 is the promoter/activator of pro-MMP-2 to active MMP-2. In mammary epithelial cell models, the overexpression of HER-2 increased the production of MMP-2, upregulating the transcription and activity of the MMP-2 promoter via MAPK and P13K [28]. Thus these mechanisms would explain our findings that, after androgen blockade, increases in HER-2-positive patients are seen, which in turn leads to stimulation of MMP-2 expression in the micrometastasis.

Stromal cells do not express HER-2, and the expression of MMP-2 in hormone naïve patients was also negative. In the groups with >6 years of androgen blockade the expression of stromal MMP-2 increases rapidly and significantly, which suggests that micrometastatic MMP-2 expression may in some way activate stromal MMP-2 expression; in other words “the seed” modulates “the soil.” The increased expression of stromal MMP-2 would increase the neovascularization and thus support rapid tumor growth.

In summary, although our study has the limitation of being cross-sectional and lacking changes of phenotypic expression with time, it would appear that with androgen blockade there is early selection of HER-2 positive cancer cells; HER-2 activates the androgen receptor through MAPK and/or AKT pathways leading to androgen independence and increased expression of MMP-2 promoter and MMP-2 activity in the micrometastasis through the same pathways. This increased MMP-2 activity by way of soluble factors and/or direct contact leads to activation of pro-MMP-2 in the stroma and thus could promote angiogenesis and tumor growth. The results suggest that in selected patients the addition of anti-HER-2 therapy to androgen blockade may be of benefit. The use of bisphosphonates as nonselective anti-metalloproteinase-2 agents may also have a possible role in these patients.

## Figures and Tables

**Table 1:** Detection of micrometastasis (mM) according to Gleason score and stage.

Gleason score	4	5	6	7	8 + 9	Total
No. mM/total patients	1/5*	14/17*	7/9	3/3	1/1	26/35

Stage	1	2	3			Total
No. mM/total patients	3/9**	13/15**	10/11			26/35

^∗,∗∗^ *P* < 0.02 Fisher 2 tailed.

**Table 2:** Detection of micrometastasis (mM) according to Gleason score and stage.

Gleason score	4	5	6	7	8 + 9	Total
No. mM/total patients	2/4	6/10	8/12	9/12	3/5	28/43

Stage	1	2	3			Total
No. mM/total patients	0/1	7/15*	21/27*			28/43

*P < 0.04 Chi-squared.

**Table 3:** Coexpression of MMP-2 and HER-2 in micrometastasis.

	HER-2 (+)	HER-2 (−)	Total
MMP-2 (+)	16	5	21
MMP-2 (−)	25	41	66

Total	41	46	87

*P* = 0.005, Chi-squared.

**Table 4:** Coexpression of MMP-2 and HER-2 according to serum PSA levels.

Serum PSA	MMP-2 (+) HER-2 (+)	MMP-2 (+) HER-2 (−)	MMP-2 (−) HER-2 (+)	MMP-2 (−) HER-2 (−)	Total
<2.0 ng/mL	2	0	12	26	40
2–10 ng/mL	2	2	9	8	21
>10.0 g/mL	13	2	2	9	26

Total	17	4	23	43	87

**Table 5:** Comparison of MMP-2 and HER-2 coexpression with time.

Years of treatment	MMP-2 (+) HER-2 (+)	MMP-2 (+) HER-2 (−)	MMP-2 (−) HER-2 (+)	MMP-2 (−) HER-2 (−)	Total
0–2	1	3	10	19	33
3–5	6	0	9	10	25
6–10	7	1	4	10	22
>10	3	0	0	4	7

Total	17	4	23	43	87

**Table 6:** Comparison of stromal and micrometastasis (mM) MMP-2 expression.

	mM MMP-2 (+)	mM MMP-2 (−)	Total
Stromal MMP-2 (+)	13	1	14
Stromal MMP-2 (−)	8	65	73

Total	21	66	87 *P* < 0.00001

**Table 7:** Comparison of stromal and micrometastasis (mM) HER-2 expression.

	mM HER-2 (+)	mM HER-2 (−)	Total
Stromal MMP-2 (+)	14	0	14
Stromal MMP-2 (−)	27	46	71

Total	41	46	87 *P* < 0.0005

**Table 8:** Stromal expression of MMP-2 compared with serum PSA.

PSA	Stromal MMP-2 (+)	Stromal MMP-2 (−)	Total
<2.0 ng/mL	0	40	40
2.0–10.0 ng/mL	3	18	21
>10.0 ng/mL	11	15	26

Total	14	73	87

**Table 9:** Comparison of stromal MMP-2 expression with time.

	Stromal MMP-2 (+)	Stromal MMP-2 (−)	Total
0–2 years	0	33	33
3–5 years	1	24	25
6–10 years	7	15	22
>10 years	6	1	7

Total	14	73	87
